# Bicuculline Reduces Neuroinflammation in Hippocampus and Improves Spatial Learning and Anxiety in Hyperammonemic Rats. Role of Glutamate Receptors

**DOI:** 10.3389/fphar.2019.00132

**Published:** 2019-02-25

**Authors:** Michele Malaguarnera, Marta Llansola, Tiziano Balzano, Belén Gómez-Giménez, Carles Antúnez-Muñoz, Núria Martínez-Alarcón, Rahebeh Mahdinia, Vicente Felipo

**Affiliations:** ^1^Laboratory of Neurobiology, Centro Investigación Príncipe Felipe de Valencia, Valencia, Spain; ^2^Faculty of Biology, Damghan University, Damghan, Iran

**Keywords:** hepatic encephalopathy, GABA_A_ receptor, hippocampus, spatial learning and memory, anxiety, glutamate receptors, astrocytes activation, IL-1β

## Abstract

Patients with liver cirrhosis may develop minimal hepatic encephalopathy (MHE) with mild cognitive impairment. Hyperammonemia is a main contributor to cognitive impairment in MHE, which is mediated by neuroinflammation. GABAergic neurotransmission is altered in hyperammonemic rats. We hypothesized that, in hyperammonemic rats, (a) enhanced GABAergic tone would contribute to induce neuroinflammation, which would be improved by reducing GABAergic tone by chronic bicuculline treatment; (b) this would improve spatial learning and memory impairment; and (c) modulation of glutamatergic neurotransmission would mediate this cognitive improvement. The aim of this work was to assess the above hypotheses. Bicuculline was administrated intraperitoneally once a day for 4 weeks to control and hyperammonemic rats. The effects of bicuculline on microglia and astrocyte activation, IL-1β content, on membrane expression of AMPA and NMDA glutamate receptors subunits in the hippocampus and on spatial learning and memory as well as anxiety were assessed. Treatment with bicuculline reduces astrocyte activation and IL-1β but not microglia activation in the hippocampus of hyperammonemic rats. Bicuculline reverses the changes in membrane expression of AMPA receptor subunits GluA1 and GluA2 and of the NR2B (but not NR1 and NR2A) subunit of NMDA receptors. Bicuculline improves spatial learning and working memory and decreases anxiety in hyperammonemic rats. In hyperammonemia, enhanced activation of GABA_A_ receptors in the hippocampus contributes to some but not all aspects of neuroinflammation, to altered glutamatergic neurotransmission and to impairment of spatial learning and memory as well as anxiety, all of which are reversed by reducing activation of GABA_A_ receptors with bicuculline.

## Introduction

Patients with liver cirrhosis may develop covert or MHE with mild cognitive impairment, attention deficits and psychomotor slowing, which impair quality of life, reduce life span and increase accidents, falls and hospitalizations. MHE affects several million people and is a serious health, social and economic problem (Felipo, 2013).

Hyperammonemia and peripheral inflammation play synergistic roles in inducing the cognitive and motor alterations in MHE (Shawcross et al., 2004; Montoliu et al., 2009; Felipo et al., 2012). These alterations would be mediated by neuroinflammation (Montoliu et al., 2015).

Chronic hyperammonemia *per se* is enough to induce neuroinflammation with activation of microglia and increased inflammatory markers in the brain associated with impaired cognitive function (Rodrigo et al., 2010). Reducing neuroinflammation with ibuprofen restores learning in a Y maze task in hyperammonemic rats (Rodrigo et al., 2010). Hyperammonemic rats also show neuroinflammation in the hippocampus that is associated with altered membrane expression of glutamate receptors and impaired spatial learning and memory (Cabrera-Pastor et al., 2016). These alterations are reversed by treating the rats with sulforaphane, which reduces neuroinflammation in the hippocampus (Hernández-Rabaza et al., 2016).

GABAergic neurotransmission is altered in hyperammonemic rats, which show increased GABAergic tone in the cerebellum. Chronic treatment with bicuculline, a GABA_A_ receptor antagonist, restores GABAergic tone, the function of the glutamate-nitric oxide-cGMP pathway in the cerebellum and learning of a discrimination task modulated by this pathway (Cauli et al., 2009). The same effects are induced by treatment with pregnenolone sulfate, a negative allosteric modulator of the GABA_A_ receptor, which also improves motor incoordination caused by increased extracellular GABA in the cerebellum (González-Usano et al., 2013).

Reducing GABAergic tone by treating rats with GR3027, which antagonizes the enhancement of GABA_A_ receptor activation by neurosteroids, also restores spatial memory modulated mainly in the hippocampus (Johansson et al., 2015).

These reports show that reducing either GABAergic tone or neuroinflammation in the hippocampus of hyperammonemic rats improve spatial learning. This suggests that there would be a cross-talk between GABAergic tone and neuroinflammation in the modulation of the mechanisms involved in spatial learning and maybe also in other functions modulated in the hippocampus such as short-term memory or anxiety.

Recent studies support this cross-talk between GABAergic neurotransmission and neuroinflammation, which seem to modulate each other (reviewed by Crowley et al., 2016). Different pro-inflammatory cytokines, such as TNFα, IL-1β and IL-6, modulate GABA_A_ receptor function in an area- and dose-dependent manner (Stellwagen et al., 2005; García-Oscos et al., 2012; Pribiag and Stellwagen, 2013). IL-1β suppresses GABA-induced currents in the superficial spinal cord (Kawasaki et al., 2008) in hippocampal slices (Nisticò et al., 2013) and in rat hippocampal neurons (Wang et al., 2000). Contrarily, at a different concentration, IL-1β also increases membrane expression of GABA_A_ receptor subunits and GABAergic neurotransmission in cultured rat hippocampal neurons (Serantes et al., 2006). Hellstrom et al. (2005) showed that LPS increases GABAergic inhibition in the hippocampus through IL-1β. Additionally, reactive astrocytes release GABA, increasing GABAergic tone in cerebral ischemia (Lin et al., 2018). GABA released from reactive astrocytes impairs learning and memory (Jo et al., 2014). During neuroinflammation, GABAergic tone would increase to reduce excitotoxicity caused by excessive glutamate neurotransmission (Crowley et al., 2016).

In rats with hyperammonemia or hepatic encephalopathy, reducing neuroinflammation reverses the increase of GABAergic tone in the cerebellum and restores impaired motor coordination, suggesting enhancement of GABA neurotransmission by neuroinflammation in the cerebellum in these rats (Rodrigo et al., 2010; Dadsetan et al., 2016a; Hernández-Rabaza et al., 2016; Agusti et al., 2017). These reports show that neuroinflammation modulates GABAergic neurotransmission in different systems, including the cerebellum of hyperammonemic rats.

Gamma-aminobutyric acid neurotransmission also modulates neuroinflammation. Both anti- and pro-inflammatory effects of GABA have been reported. GABA acts as anti-inflammatory in rheumatoid arthritis, downregulating mechanisms that lead to the production of pro-inflammatory agents such as IL-1β (Kelley et al., 2008) and also in neuroinflammation in general (Crowley et al., 2016). GABA acts as anti-inflammatory in microglia through activation of GABA_A_ receptors (Lee et al., 2011).

Other studies suggest that GABA can induce pro-inflammatory cytokines in pathological conditions. Carmans et al. (2013) showed that exogenous GABA increases IL-6 and TNFα mRNA in the CNS. Sallam et al. (2016) reported that intra-cerebral administration of bicuculline inhibited the increase of IL-6 and TNFα induced by LPS in rats. Increased GABA levels and the subsequent activation of GABA_A_ receptors induce activation of astrocytes (Runquist and Alonso, 2003).

The mechanisms by which neuroinflammation impairs spatial learning in hyperammonemic rats involve altered membrane expression of AMPA and NMDA receptor subunits. Treatment with sulforaphane reverses changes in membrane expression of the receptors and restores spatial learning (Hernández-Rabaza et al., 2016).

It has not been analyzed whether modulating GABAergic neurotransmission could reduce neuroinflammation in the hippocampus of hyperammonemic rats and restore membrane expression of glutamate receptors and cognitive functions modulated by this area.

We have proposed that there is an interplay between neuroinflammation and GABAergic-glutamatergic neurotransmission in the induction of cognitive and motor alterations in rats with MHE (Agusti et al., 2017). We propose now that a similar interplay in the hippocampus of hyperammonemic rats would induce alterations in spatial learning and is likely in other functions modulated in the hippocampus, such as anxiety or short-term memory.

We hypothesized that, in hyperammonemic rats, (a) enhanced GABAergic tone would contribute to induce neuroinflammation in hippocampus; (b) reducing GABAergic tone by chronic treatment with bicuculline would reduce neuroinflammation; (c) this would be associated with normalization of membrane expression of AMPA receptor subunits and restoration of spatial learning; and (d) bicuculline treatment could also improve other functions modulated in the hippocampus such as short-term-memory and anxiety.

To test these hypotheses, we assessed whether chronic intraperitoneal administration of the GABA_A_ receptor antagonist bicuculline in hyperammonemic rats modulates neuroinflammation in the hippocampus by analyzing the activation of microglia, astrocytes, and IL-1β content. We also assessed the effects on membrane expression of AMPA and NMDA receptor subunits and on spatial learning and memory, short-term memory and anxiety.

## Materials and Methods

### Study Design, Chronic Hyperammonemia in Rats, and Treatment With Bicuculline

Male Wistar rats (120–140 g, Charles River Laboratories, Barcelona, Spain) were made hyperammonemic by feeding them an ammonium-containing diet as previously described (Felipo et al., 1988). Animals were distributed into four groups: control with vehicle (CV); control treated with bicuculline (CB); hyperammonemic rats (HA); hyperammonemic rats treated with bicuculline (HB). Bicuculline [(+)-Bicuculline, Sigma-Aldrich] was injected intraperitoneally at 0.3 mg/kg once per day. The dose was chosen based on a previous study that shows that hyperammonemia changes GABAergic tone in the CNS (Cauli et al., 2009). This dose is lower than that which induces seizures (>1 mg/kg i.p.) (Meldrum et al., 1987; Giardina, 2000; Martín del Campo et al., 2009). In addition, we did not observe any signs of seizures in any of the injected rats. Bicuculline was dissolved in physiological serum (NaCl 0.9%) with 0.3% DMSO, and this solution was used as vehicle.

**FIGURE 1:**
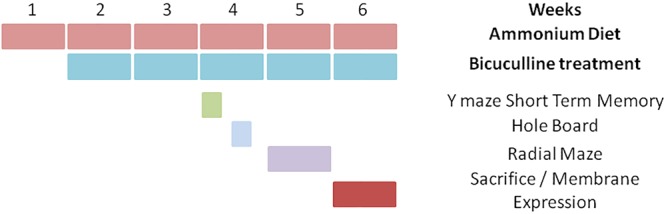
Scheme showing the experimental design.

The experiment was replicated four times using 36 animals (9 rats per group) each time. A total of 144 rats were used, with 36 rats per group. Not all animals performed behavioral tests because we found that the data obtained with two replicates were enough to reach statistical significance. Concerning the analysis of neuroinflammation and membrane expression of proteins, in each replicate four rats per group were perfused for immunohistochemistry studies while the other five rats per group were used for analysis of membrane surface expression and content of the proteins. The experimental design is summarized in Figure 1. The experiments were approved by the Comité de Ética y Bienestar en Experimentación Animal, Prince Felipe Research Center-Consellería de Agricultura, Generalitat Valenciana and carried out in accordance with the Directive of the European Commission (2010/63/EU) for care and management of experimental animals.

### Brain Immunohistochemistry

At week 6 of hyperammonemia, the rats were anesthetized with sodium pentobarbital and transcardially perfused with 0.9% saline followed by 4% paraformaldehyde in 0.1 M phosphate buffer (pH 7.4). Brains were removed and post-fixed in the same fixative solution for 24 h at 4°C. Paraffin-embedded sections (5 μm) were cut and mounted on coated slide glass. The tissue sections were then processed with the Envision Flex+kit (DAKO) blocking endogenous peroxidase activity for 5 min and then incubated with antibodies. Primary antibodies were against Iba-1 (Wako 019-19741; 1:300 for 30 min), GFAP (Dako IR524; ready for use for 20 min) and IL-1β (Abcam AB9722; 1:100 dilution for 30 min). The reaction was visualized by incubation with Envision Flex + horseradish peroxidase for 20 min and finally diaminobenzidine for 10 min. Sections were counterstained with Mayer’s hematoxylin for 5 min.

### Analysis of Microglia Activation

Analysis of Iba-1-stained microglia was performed in the hippocampus using Image J software. Microglia activation was assessed by measuring the cell perimeter in eight randomly selected areas (0.45 mm^2^) per section according to Vinet et al. (2012). The area of interest was selected. Using Auto Local Threshold and Analyze particle functions in ImageJ, the intensity thresholds and size filter were applied. To measure the perimeter of microglia, the Bernsen method was used and a 2000–20,000 size filter was applied. For each rat, at least 30–40 cells were quantified, and the results were converted from pixels to micrometers. The perimeter length for each group is expressed as the percentage of values for control rats.

### Analysis of Astrocytes Activation

Astroglial area (μm^2^) covered by GFAP was measured using Image J software. Using Auto Local Threshold and Analyze Particles functions, the intensity thresholds and size filter were applied. To measure the total cell size, the Bernsen method (Bernsen, 1986) was used and a 1500–7000 size filter was applied. For each rat, at least 20 cells from three different sections were counted.

### Analysis of IL1β Expression

IL1β-positive cells were manually counted by two blinded experimenters and the results (the mean of two blind experimenters) were expressed as a percentage of the total number of cells. For each rat, at least 120–150 cells per section were counted from at least four different sections. The intensity of IL1β in CA1 region was quantified using the ROI manager function in ImageJ. The CA1 region was selected manually. Inverted values of Mean Gray value were recorded and results were expressed as a percentage of control group.

### Analysis of Membrane Surface Expression of Receptors

This analysis was performed by cross-linking with B53 as described by Cabrera-Pastor et al. (2016). Hippocampi were dissected and transversal slices (400 μm) were obtained using a chopper. Slices were added to tubes containing ice-cold standard buffer with or without 2 mM BS3 (Pierce, Rockford, IL, United States) and incubated for 30 min at 4°C. Cross-linking was terminated by adding 100 mM glycine (10 min, 4°C). The slices were homogenized by sonicating for 20 s. Samples treated or not with BS3 were analyzed by western blot. The membrane surface expression of each receptor was calculated as the difference between the intensity of the bands without BS3 (total protein) and with BS3 (non-membrane protein) as described by Cabrera-Pastor et al. (2016).

### Analysis of Protein Content in Hippocampus by Western Blot

Homogenates of the hippocampus were subjected to immunoblotting according to Felipo et al. (1993). Primary antibodies were against IL-1β 1:500 dilution (AF-510-NA) from R&D SYSTEMS (Minneapolis, MN, United States); GluA1, GluA2, NR2A, and NR2B 1:1000 dilution (cat.# 04-855, AB1768, 04-901 and 06-600, respectively) from Merck Millipore (Darmstadt, Germany); and NR1, 1:1000 dilution (cat.# 556308) from BD Biosciences (San Jose, CA, United States). As a control for protein loading, the same membranes were also incubated with anti-actin (1:5000) from Abcam (Cambridge, MA, United States). Secondary antibodies were anti-rabbit, anti-goat or anti-mouse IgG, 1:4000 dilution (cat.# A8025, A7650, A3562, respectively) conjugated with alkaline phosphatase from Sigma (St. Louis, MO, United States). The images were captured using the ScanJet 5300C (Hewlett-Packard, Amsterdam, Netherlands) and band intensities quantified using the A Imager 2200, version 3.1.2 (AInnotech Corporation, San Francisco, CA, United States).

### Spatial Learning in the 8-Arms Radial Maze

It was assessed as described in Hernández-Rabaza et al. (2016). Training was performed over 4 days (three trials per day). The task involved locating four pellets, each placed at the end of a different arm according to a random configuration. Configurations were specific for each rat and were kept invariable throughout training. The number of spatial reference errors (reference memory errors, visits to unbaited arms) and WM errors (WM errors, visits to arms already visited in the same trial) were calculated. Learning index is defined as the difference between the number of right choices and reference errors as in Hernández-Rabaza et al. (2016).

### Short-Term Spatial Recognition Memory

It was analyzed using a Y-maze consisting of three arms made of black metacrilate joined in the middle to form a “Y” shape. This test is based on the rodents’ innate curiosity to explore novel areas and presents no negative or positive reinforcement and very little stress for the rats. The protocol is a modification of the test used by Sarnyai et al. (2000) and Sanderson et al. (2009). The rat was handled for 1 min to reduce stress and anxiety, placed into one of the arms of the maze (start arm) and allowed to explore the maze with one of the arms closed for 2 min (training trial) for three times. After 1 min of inter-trial interval, the rat returned to the Y maze by placing it in the start arm. Then, the rat was allowed to explore freely all three arms of the maze for 2 min (test trial). The number of entries into and the time spent in each arm, the first choice of entry and the discrimination ratio [(Time spent in the novel arm – Time spent in the familiar arm)/Total time passed in the two arms] were registered. Because entry into the novel arm could be altered by anxiety, exploration time was also recorded. No significant differences in exploration time were observed between groups (results not shown).

### Analysis of Anxiety Using a Hole-Board Test

This test was performed in a plastic floor with 16 equidistant holes of 4 cm in diameter. The floor was positioned in an open-field activity chamber (43 cm × 43 cm × 30.5 cm) (Med Associates, St Albans, VT, United States), where the animals were positioned. The rat’s activity was detected by arrays of infrared motion detection, with two arrays 1 cm above the floor of the chamber and another array 6 cm above the floor, so the upper line of infra-red cells detected the animal movement and the bottom line detected the head-dipping. For the hole-board experiments, each animal was placed in the center of the hole-board and allowed to freely explore the apparatus for 5 min (Kong et al., 2006). Total number of head-dipping and latency to the first head-dipping were recorded by the software Activity Monitor (provided by MED Associates, Inc., St Albans, VT, United States). Anxiety leads to decreased explorative activity that can be quantified by counting the frequency of head-dipping in the holes. The larger is the frequency of head-dipping and the lower is the anxiety of the rat. Novel head dips were also measured to quantify anxiety.

### Statistical Analysis

Data are expressed as mean ± SEM. All statistical analyses were performed using the software program GraphPad Prism 7.0 (GraphPad Prism Software, Inc.). Statistical analysis was carried out using one-way ANOVA or two-way ANOVA with repeated measures, followed by Tukey’s *post hoc* test, as indicated in the figure legends. A confidence level of 95% was accepted as significant.

## Results

**FIGURE 2:**
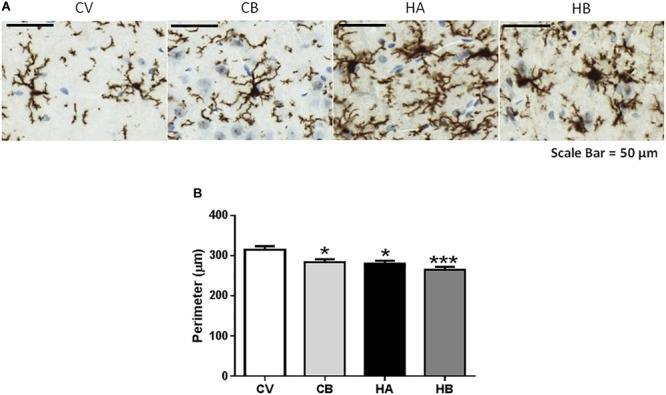
Effect of bicuculline on microglia activation in CA1 region of the hippocampus. Immunohistochemistry was performed as indicated in Section “Materials and Methods” using antibody against Iba1. Representative images of microglial activation **(A)** are shown. Scale bar = 50 um. The perimeter of microglia stained with Iba1 was quantified as described in methods as a measure of microglia activation. **(B)** Data are the mean ± SEM of 10 rats per group. Values significantly different from control rats are indicated by asterisks. ^∗^*p* ≤ 0.05. CV, control vehicle; CB, control treated with bicuculline; HV, hyperammonemic rats with vehicle; HB, hyperammonemic rats treated with bicuculline. ^∗∗∗^*p* < 0.001.

Hyperammonemic rats show activated microglia in the hippocampus, with a less ramified and more ameboid morphology reflected in a reduction (*p* < 0.05) of the perimeter to 280 ± 8 μm compared to 315 ± 9 μm in control rats (Figure 2). Activation of microglia is not prevented by bicuculline. The perimeter of microglia in hyperammonemic rats treated with bicuculline was 265 ± 7 μm, similar to untreated hyperammonemic rats. In contrast, bicuculline treatment induced microglia activation in control rats, with reduction of the perimeter to 284 ± 7 μm [*F*(3,68) = 12,76] (Figure 2).

**FIGURE 3:**
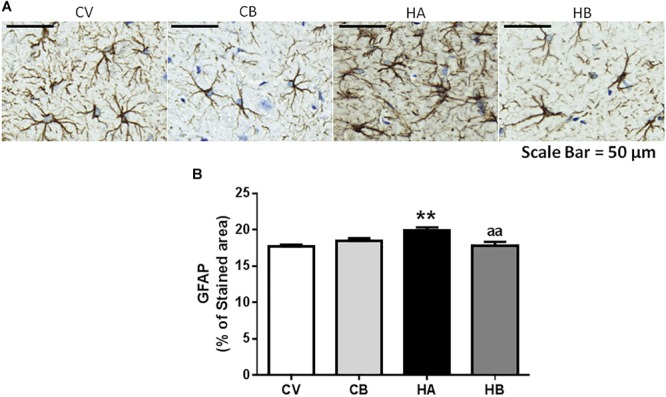
Effect of bicuculline on astrocyte activation in CA1 region of hippocampus. Immunohistochemistry was performed as indicated in Section “Materials and Methods” using antibody against GFAP. Representative images of astrocyte staining **(A)** are shown. Scale bar = 50 um. The percentage of the area stained with GFAP was quantified as described in methods **(B)**. Data are the mean ± SEM of 10 rats per group. Values significantly different from control rats are indicated by asterisks. ^∗∗^*p* ≤ 0.01. Values significantly different from hyperammonemic rats are indicated by “aa” *p* ≤ 0.01. CV, control vehicle; CB, control treated with bicuculline; HV, hyperammonemic rats with vehicle; HB, hyperammonemic rats treated with bicuculline.

Hyperammonemic rats also showed astrocyte activation in the hippocampus, as indicated by the increased area stained by anti-GFAP (19.9 ± 0.4% of the area, *p* < 0.01) compared to control rats (17.7 ± 0.2% of the area). Bicuculline decreased astrocyte activation in hyperammonemic rats and the area stained by anti-GFAP returned to normal values (17.8 ± 0.5% of the area, *p* < 0.01 vs. hyperammonemic rats), indicating de-activation of astrocytes [*F*(3,56) = 6,068] (Figure 3).

**FIGURE 4:**
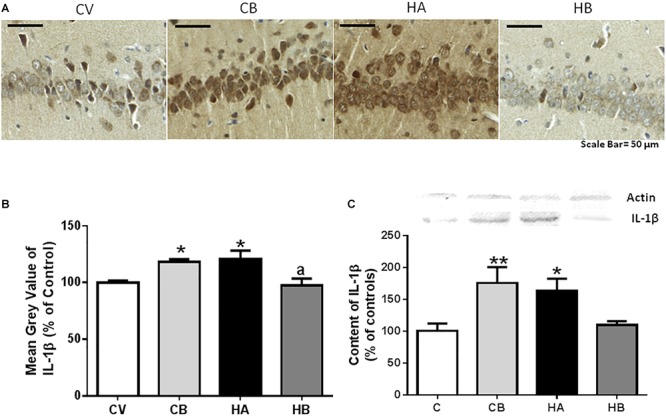
Effect of bicuculline on the content of IL1β in CA1 region of hippocampus. Immunohistochemistry was performed as indicated in Section “Materials and Methods” using antibody against IL1β. Representative images of IL1β staining in CA1 are shown **(A)**. Scale bar = 50 um. The percentage of cells expressing IL1β was quantified as described in methods. Data are the mean ± SEM of 10 rats per group **(B).** The content of IL-1 β was also analyzed by western blot in the total hippocampus **(C)**. Values are mean ± SEM of 12–14 samples per group. Values significantly different from control rats are indicated by asterisks, ^∗^*p* ≤ 0.05, ^∗∗^*p* < 0.01. Values significantly different from hyperammonemic rats are indicated by “a” *p* ≤ 0.05. CV, control vehicle; CB, control treated with bicuculline; HV, hyperammonemic rats with vehicle; HB, hyperammonemic rats treated with bicuculline.

Neuroinflammation was also reflected in increased IL1β content in hippocampus as analyzed by immunohistochemistry. Anti-IL1β stained mainly neurons of the CA1 region. The number of cells expressing IL1β increased (*p* < 0.05) in hyperammonemic rats to 120 ± 8% of controls. Treatment with bicuculline reversed the increase in IL1β to 97 ± 12% of controls (*p* < 0.05, compared with untreated hyperammonemic rats). In contrast, as occurs for microglial activation, bicuculline increased (*p* < 0.05) the number of cells expressing IL1β in control rats to 118 ± 3% of untreated controls [*F*(3,42) = 5,384] (Figure 4A,B).

IL1β was also quantified by western blot, which confirmed an increase of IL1β levels in the hippocampus of hyperammonemic rats to 159 ± 2% of control rats. This increase was reversed by bicuculline treatment, which reduces IL1β levels to 97 ± 12% of controls (Figure 4C). Western blot analysis also confirmed the increase (*p* < 0.05) of IL1β in control rats treated with bicuculline, to 189 ± 32% of untreated controls [*F*(3,30) = 5,427] (Figure 4C).

We have proposed that neuroinflammation in the hippocampus leads to impairment of spatial learning and memory by altering membrane expression of GluA1 and GluA2 subunits of AMPA receptors (Cabrera-Pastor et al., 2016). We therefore assessed if treatment with bicuculline normalizes membrane expression of GluA1 and GluA2 subunits of AMPA receptors and spatial learning and memory in hyperammonemic rats.

**FIGURE 5:**
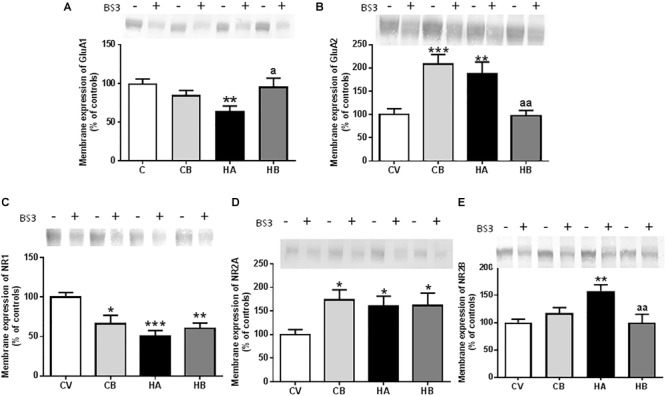
Effect of bicuculline on membrane expression of AMPA and NMDA receptors subunits. Membrane expression of GluA1 **(A)**, GluA2 **(B)**; NR1 **(C)**, NR2A **(D)**, and NR2B **(E)** in the hippocampus was analyzed using the BS3 crosslinker procedure as described in Section “Materials and Methods.” Samples incubated in the absence or presence of BS3, were subjected to Western blotting using antibodies for each of the subunits. Representative images are shown. Samples in the absence of BS3 represent the total amount of each protein. Samples in the presence of BS3 represent the non-membrane fraction. The intensities of the bands were quantified and membrane expression was calculated as the difference of intensity between samples without and with BS3. Values are expressed as a percentage of control rats and are the mean ± standard errors of 8–10 rats per group. Values significantly different from control rats are indicated by asterisks and from hyperammonemic rats by “a.” ^∗^*p* < 0.05; ^∗∗^*p* < 0.01; ^∗∗∗^*p* < 0.001; a *p* < 0.05; aa *p* < 0.01.

At 5 weeks of hyperammonemia, membrane expression of GluA1 was significantly reduced (*p* < 0.01) to 65 ± 6% of control rats (Figure 5A). Treatment with bicuculline reversed the decrease in membrane expression of GluA1 (*p* < 0.01 compared with hyperammonemic rats), returning to 96 ± 11% of control rats. In control rats, treatment with bicuculline induced a slight reduction (85 ± 6% of control rats) in membrane expression of GluA1 [*F*(3,47) = 5,667] (Figure 5A).

Membrane expression of GluA2 was increased (*p* < 0.01) in hyperammonemic rats to 188 ± 25% of control rats (Figure 5B). Bicuculline completely reversed the increase of GluA2 (*p* < 0.01, compared with untreated hyperammonemic rats), returning to levels similar to controls (97 ± 12% of controls). In control rats, bicuculline increased membrane expression of GluA2 (208 ± 21% of controls, *p* < 0.001) [*F*(3,47) = 10,74] (Figure 5B).

We also assessed the effects on the NR1 and NR2 subunits of NMDA receptors. Membrane expression of NR1 was significantly reduced [*p* < 0.001; *F*(3,58) = 7,592] in hyperammonemic rats to 50 ± 7% of controls (Figure 5C). Membrane expression of NR2A was increased [*p* < 0.05; *F*(3,49) = 3,752] to 161 ± 21% of control rats (Figure 5D), and that of NR2B to 158 ± 11% of control rats [*p* < 0.01; *F*(3,53) = 6,066] (Figure 5E). Treatment with bicuculline in hyperammonemic rats did not reverse the decrease of NR1 nor the increase in NR2A, but completely normalized (*p* < 0.01) the membrane expression of NR2B, returning it to levels similar to control rats (Figure 5C–E).

In control rats, bicuculline reduced (*p* < 0.05) membrane expression of NR1 to 66 ± 11% of controls and increased membrane expression of NR2A (173 ± 21% of controls, *p* < 0.05) (Figure 5C,D).

**FIGURE 6:**
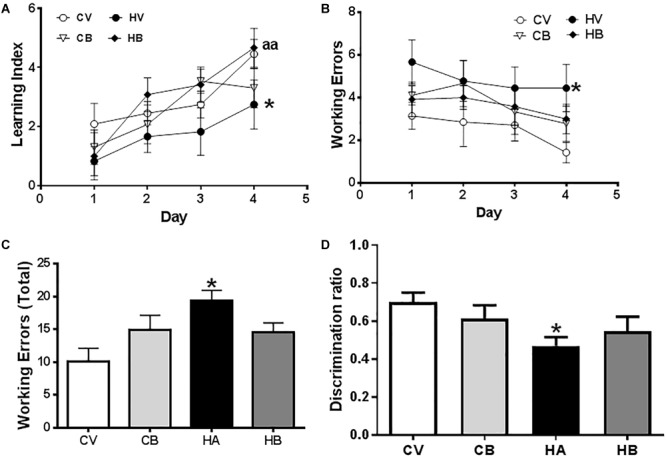
Effects of bicuculline on spatial learning and WM. Spatial learning and memory were assessed in the radial maze. A learning index was calculated as described in Section “Materials and Methods.” Learning index increases along the training days **(A)**. **(B)** Working errors at the different days of training. **(C)** Total working errors in the total period of 4 days. Short-term memory in the Y maze was assessed as described in Section “Materials and Methods” and the discrimination index of each experimental group is shown in **(D)**. Values are the mean ± SEM of 12–13 rats per group. Values significantly different from control rats are indicated by asterisks and from hyperammonemic rats by “a.” ^∗^*p* < 0.05; aa *p* < 0.01.

Spatial learning and memory were assessed at 4–5 weeks of hyperammonemia in the radial and Y mazes. Hyperammonemia impaired spatial learning in the radial maze. The learning index was lower (*p* < 0.05) than for control rats. Bicuculline reversed the impairment of spatial learning in hyperammonemic rats. The learning index was higher than in untreated hyperammonemic rats [two-way ANOVA Repeated Measure: group by Learning Index. *F*(3,44) = 3,684, *p* < 0.01] and not different from control rats (Figure 6A).

The number of working errors was also higher in hyperammonemic than in control rats [two-way ANOVA: group by treatment interaction *F*(3,33) = 3,706, *p* < 0.05] (Figure 6B). Hyperammonemic rats performed a total of 19 ± 2 working errors [*F*(3,33) = 3.7], which is more (*p* < 0.05) than for control rats (10 ± 2 working errors). Treatment with bicuculline reversed the impairment of WM. Hyperammonemic rats treated with bicuculline performed 15 ± 2 working errors, not significantly different from control rats. In control rats, bicuculline increased the number of working errors (15 ± 2), but the difference with untreated controls was not statistically significant (Figure 6C).

We also assessed short-term memory in the Y maze. The discrimination ratio was reduced (*p* < 0.05) in hyperammonemic rats to 0.47 ± 0.05, compared with control rats (0.70 ± 0.05) [*F*(3,43) = 2,6]. Treatment with bicuculline partially restored short-term memory. The discrimination ratio (0.55 ± 0.08) was higher than in untreated hyperammonemic rats (Figure 6D).

**FIGURE 7:**
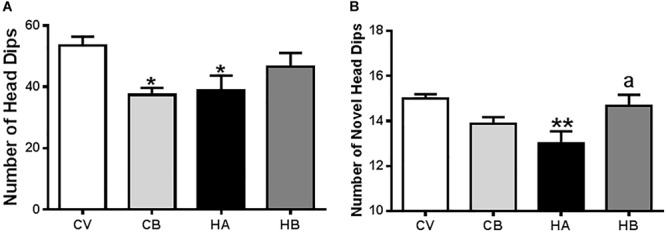
Effects of bicuculline on anxiety. Anxiety was assessed using a hole-board test in an actimeter as indicated in Section “Materials and Methods.” Total and novel head dips were quantified by the Activity monitor software and the means ± SEM of eight rats per group are represented in **(A,B)**, respectively. Values significantly different from control rats are indicated by asterisks and from hyperammonemic rats by “a.” ^∗^*p* < 0.05, ^∗∗^*p* < 0.01; a *p* < 0.05.

As anxiety is also modulated by the hippocampus, we also assessed it in hyperammonemic rats, which show a decreased number of head dips [39 ± 5, *p* < 0.05, *F*(3,25) = 4,548] compared to control rats (55 ± 5), indicative of anxiety behavior. Hyperammonemic rats treated with bicuculline showed a tendency to increase the number of head dips, reaching 47 ± 5, which is not significantly different from control rats (Figure 7A).

Control rats treated with bicuculline showed a similar number of head dips (37 ± 2, *p* < 0.05) than hyperammonemic rats (Figure 7A), which is not surprising since bicuculline has a well-known anxiogenic effect.

We also analyzed the number of novel head dips, i.e., the number of head dips in unexplored holes. This number was reduced (*p* < 0.01) in hyperammonemic rats (13.0 ± 0.5) compared to control rats (15.3 ± 0.2) and was restored by treatment with bicuculline [15.1 ± 0.5; *F*(3,25) = 5,482] (Figure 7B). In control rats, treatment with bicuculline reduced the number of novel head dips (14.0 ± 0.3), indicating altered exploratory behavior.

## Discussion

We have analyzed the effects of blocking GABA_A_ receptors with bicuculline on three indicators of neuroinflammation in the hippocampus of hyperammonemic rats: activation of microglia and of astrocytes and content of IL1β. Blocking GABA_A_ receptors reduces astrocyte activation and IL1β levels, but not microglia activation in the hippocampus of hyperammonemic rats.

GABA_A_ receptors are expressed in astrocytes (MacVicar et al., 1989; Bureau et al., 1995) and their expression seems to be increased in reactive astrocytes (Hösli et al., 1997).

Moreover, it has been shown in primary cultures that GABA released by neurons or added exogenously triggers morphological changes in astrocytes through the activation of GABA_A_ receptors. The effect was blocked by bicuculline (Matsutani and Yamamoto, 1997). A similar effect of GABA on astrocytes morphology through activation of GABA_A_ receptors has been shown *in vivo* in neonatal rat hypothalamus (Mong et al., 2002).

The increase of GABAergic signaling *in vivo*, by using an inhibitor of GABA transaminase or intracerebral infusion of the GABA-A agonist muscimol, induced activation of astrocytes, with increased branching and GFAP content (Runquist and Alonso, 2003).

The above reports suggest that increasing GABA levels and/or activation of GABA_A_ receptors is enough to induce activation of astrocytes. As we show that bicuculline treatment reverses activation of astrocytes in the hippocampus of hyperammonemic rats, it is likely that this de-activation of astrocytes would be due directly to the block of GABA_A_ receptors in astrocytes by bicuculline.

Bicuculline-induced de-activation of astrocytes is associated with a reduction of IL-1β in the hippocampus of hyperammonemic rats. However, IL-1β expression in hyperammonemic rats is increased in neurons of the CA1 region, not in astrocytes, in agreement with previous reports in rats with hyperammonemia and hepatic encephalopathy (Hernandez-Rabaza et al., 2015; Dadsetan et al., 2016b; Balzano et al., 2019). Therefore, the normalization of IL-1β levels by bicuculline would not occur in astrocytes.

It was reported that IL-1β and TNF-a are expressed in hippocampal neurons *in vivo* in response to lesions (Tchélingérian et al., 1996) or to pneumococcal meningitis (Izadpanah et al., 2014). *In situ* hybridization studies show that in murine pneumococcal meningitis IL-1β and TNF-a mRNA were first upregulated in astroglial cells but at 18–24 h were strongly increased in hippocampal neurons (Izadpanah et al., 2014). A similar process occurs in the hippocampus of rats with hepatic encephalopathy, leading to increased expression of IL-1β and TNF-a in neurons (Dadsetan et al., 2016b). It seems therefore that neuronal expression of IL-1β occurs in different pathological situations and would be triggered by previous expression in astrocytes.

These reports suggest therefore that increased expression of IL-1β in CA1 neurons of the hippocampus in hyperammonemic rats could be a consequence of previous activation of astrocytes. Bicuculline would reverse activation of astrocytes and this would lead to a reduced expression of IL-1β in neurons in hyperammonemic rats. Alternatively, other direct effects of blocking GABA_A_ receptors with bicuculline could be also involved. For example, it has been reported that treatment with bicuculline methiodide, a form of the GABA_A_ receptor blocker that does not cross the blood–brain barrier, decreases IL-1β in blood in a rat model of sepsis (Hsu and Liu, 2004).

In contrast to astrocyte activation and over-expression of IL-1β, which are reversed by bicuculline in hyperammonemic rats, microglial activation is not reversed. This suggests that hyperammonemic rats show increased GABAergic tone in the hippocampus, which contributes to activation of astrocytes and to over-expression of IL-1β but not to activation of microglia.

It has already been shown that hyperammonemic rats show increased GABAergic tone in the cerebellum but not in the cerebral cortex (Cauli et al., 2009). Increased GABAergic tone in the hippocampus is supported by a report from Johansson et al. (2015) showing that reducing GABAergic tone with GR3027, which antagonizes GABA_A_ receptor potentiating neurosteroids, restores spatial learning, modulated by the hippocampus, in hyperammonemc rats.

In contrast with the effects of bicuculline in hyperammonemic rats, bicuculline increases neuroinflammation in control rats, inducing microglial activation and increasing IL-1β. It has been proposed that GABA acts as an anti-inflammatory in rheumatoid arthritis downregulating mechanisms that lead to the production of pro-inflammatory agents such as IL-1β (Kelley et al., 2008) and also in neuroinflammation in general (Crowley et al., 2016). GABA acts as an anti-inflammatory in microglia through activation of GABA_A_ receptors (Lee et al., 2011). In control rats, treatment with bicuculline would prevent this anti-inflammatory effect of GABA resulting in activation of microglia and enhanced production of IL-1β.

In hyperammonemic rats, normalization of IL-1β levels and astrocyte activation in hippocampus by bicuculline is associated with normalization of membrane expression of the GluA1 and GluA2 subunits of AMPA receptors and of the NR2B subunit of NMDA receptors as well as with improvement of spatial learning. These effects would be a direct consequence of the reduction of IL-1β levels. Taoro-Gonzalez et al. (2018) have shown that in the hippocampus of hyperammonemic rats, increased IL-1β levels over-activate the IL-1 receptor, leading to activation of Src and increased membrane expression of NR2B and GluA2 in addition to reduced membrane expression of GluA1. Blocking IL-1 receptor with the endogenous antagonist IL-1Ra reverses all these changes. This indicates that the effects of bicuculline on GluA1, GluA2, and NR2B are due to the reduction of IL-1β. In contrast, this does not normalize membrane expression of NR1 and NR2A subunits of NMDA receptors, which are not modulated by IL-1β under these conditions (Lai et al., 2006).

It has been reported that altered membrane expression of AMPA receptors in the hippocampus is responsible for impairment of spatial learning and WM in hyperammonemic rats and that reversing these changes by different treatments (sulforaphane, extracellular cGMP) restores spatial learning (Cabrera-Pastor et al., 2016; Hernández-Rabaza et al., 2016). Normalization of membrane expression of AMPA receptors following normalization of IL-1β by bicuculline treatment would be therefore responsible for improvement of spatial learning and WM in hyperammonemic rats.

Our approach does not allow us to discern if membrane expression of AMPA and NMDA receptors is altered in glial cells or in neurons. However, we can assume that the alterations occur in neurons since, in contrast with other cerebral regions, astrocytes in the hippocampus apparently lack AMPA receptors, and expression of NMDA receptors in glial cells in the hippocampus remains controversial (Rose et al., 2018). Moreover, there is clear evidence of the role of neuronal AMPA and NMDA receptors in hippocampal synaptic plasticity and spatial learning (Morris and Frey, 1997; Shapiro, 2001; Baez et al., 2018).

A direct effect of the blockade of GABA_A_ receptors by bicuculline on improvement of spatial learning cannot be disregarded. Several studies report beneficial effects on spatial learning and memory of intra-CA1 or intraseptal injection of bicuculline (Roland and Savage, 2009; Torkaman-Boutorabi et al., 2013; Yousefi et al., 2013). Intraperitoneal injection of bicuculline also improves WM in aged ovariectomized rats treated with progesterone (Braden et al., 2015) and in control or chronically stressed rats (Nishimura et al., 2017). On the other hand, bicuculline infusion in the prefrontal cortex impairs working and reference memory (Auger and Floresco, 2017). This, together with increased IL-1β, could be a possible cause of the tendency to increase working errors (not significantly) in control rats treated with bicuculline.

The NR1 subunit of NMDA receptors seems to play a relevant role in short-term memory. The dentate gyrus NR1 knockout mice exhibit a selective impairment in short-term spatial memory (Niewoehner et al., 2007). Treatment with bicuculline did not restore membrane expression of NR1 nor short-term memory in hyperammonemic rats. In both cases, there was a slight tendency to improve that did not reach statistical significance. This suggests that altered membrane expression of NR1 in the hippocampus may contribute to decreased short-term spatial memory in hyperammonemic rats.

Anxiety is common in hyperammonemic cirrhotic patients and decreases quality of life (Nardelli et al., 2013; Malaguarnera et al., 2018). Hyperammonemic rats also show increased anxiety related to neuroinflammation (Luo et al., 2014). Anxiety is modulated by the hippocampus (Nasehi et al., 2011) and by NMDA and GABA receptors (Bina et al., 2014). Injection of NMDA in the ventral hippocampus induces anxiolytic effects, which are prevented by bicuculline, indicating a cross-talk between both neurotransmitter systems in the regulation of anxiety (Bina et al., 2014). In apparent contrast with this report, Barkus et al. (2010) reported that antagonists of NMDA receptors or deletion of the NR1 subunit of NMDA receptors in ventral hippocampus also have an anxiolytic effects.

We found that hyperammonemic rats show increased anxiety and reduced membrane expression of NR1 in the hippocampus. However, treatment with bicuculline reversed anxiety but not membrane expression of NR1, indicating that this would not be directly responsible for anxiety in hyperammonemci rats.

Genetically modified mice lacking the NR2B subunit in hippocampal granule and pyramidal cells in dentate gyrus and CA1, display reduced anxiety (von Engelhardt et al., 2008). As NR2B is increased in membranes in the hippocampus of hyperammonemic rats and treatment with bicuculline reverses this increase, it is possible that the changes in NR2B could contribute to anxiety in hyperammonemic rats and to its reversal by bicuculline.

**FIGURE 8:**
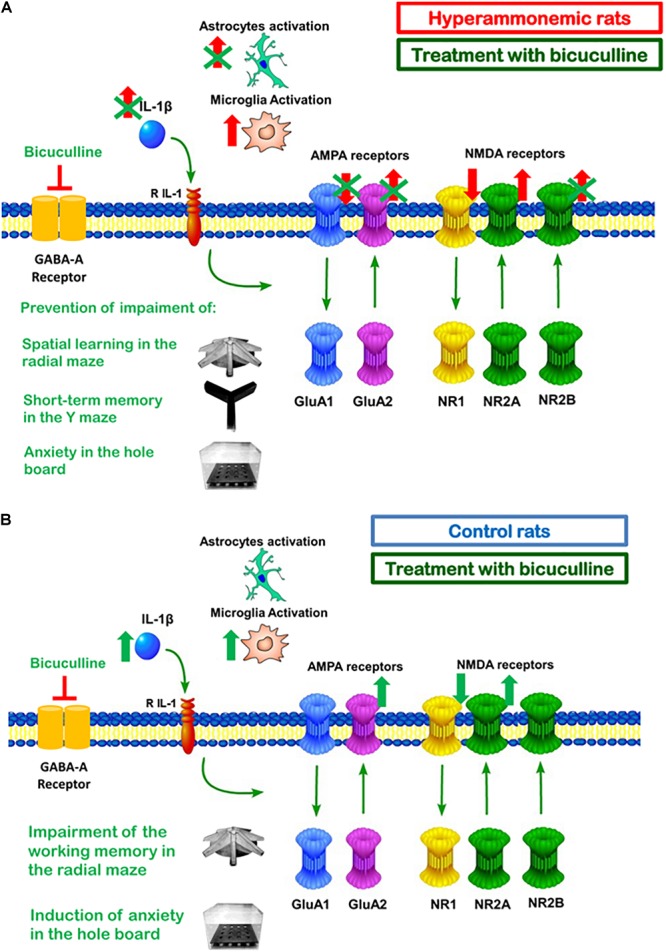
Summary of the effects of bicuculline in hippocampus of hyperammonemic **(A)** and of control **(B)** rats. **(A)** In hyperammonemic rats, treatment with bicuculline reduces astrocytes activation and IL1β expression but not microglia activation. Normalization of IL1βlevels is associated with normalization of membrane expression of the NR2B subunit of the NMDA receptor and of the GluA2 and GluA1 subunits of AMPA receptors, but not of the NR1 and NR2A subunits of the NMDA receptor. This is associated with restoration of spatial learning and decreased anxiety. **(B)** Chronic administration of bicuculline in control rats results in microglia activation and increased IL1β in the hippocampus. This is associated with increased membrane expression of GluA2 and NR2A, a tendency to increase for NR2B and reduced membrane expression of NR1. This is associated with increased anxiety and a tendency to impair WM.

As summarized in Figure 8, this report shows that in hyperammonemic rats, enhanced basal activation of GABA_A_ receptors in the hippocampus contributes to activation of astrocytes and increased IL-1β, but not to increased activation of microglia. Blocking GABA_A_ receptor with bicuculline reduces astrocyte activation and IL-1β content in hyperammonemic rats. Normalization of IL-1β levels by bicuculline is associated with reversal of the changes in membrane expression of the GluA1 and GluA2 subunits of AMPA receptors and of the NR2B subunit of NMDA receptors, but not of the NR1 and NR2A subunits of NMDA receptors. Normalization of GluA1, GluA2, and NR2B would be a consequence of normalization of IL-1β and would be responsible for restoration of spatial learning and WM. NR2B would also contribute to reverse the increased anxiety in hyperammonemic rats.

## Data Availability

All datasets generated for this study are included in the manuscript and/or the supplementary files.

## Author Contributions

MM contributed to most of the experiments including treatment of rats and obtaining samples, immunohistochemical studies and analysis, and membrane expression of receptors, performed the radial maze and anxiety tests, analyzed and interpreted the data, and drafted the manuscript. ML supervised the study, analyzed and interpreted the data, and wrote the manuscript. TB, CA-M, and RM contributed to immunohistochemical studies and analysis. BG-G contributed to treatment of rats, obtaining samples, and radial maze. NM-A contributed to membrane expression of receptors, and immunohistochemical studies and analysis. VF conceived, designed, and supervised the study, obtained funding, analyzed and interpreted the data, and wrote the manuscript.

## Conflict of Interest Statement

The authors declare that the research was conducted in the absence of any commercial or financial relationships that could be construed as a potential conflict of interest.

## Abbreviations

AMPA: alpha-amino-3-hydroxy-5-methyl-4-isoxazolepropionic acid
BS3: *Bis*(sulfosuccinimidyl) suberate
cGMP: cyclic guanosine monophosphate
CNS: central nervous system
GABA: gamma-aminobutyric acid
GFAP: glial fibrillary acidic protein
GluA: AMPA receptor subunit
IL: interleukin
IL-1R: interleikin-1 receptor
LPS: lipopolysaccharide
MHE: minimal hepatic encephalopathy
NMDA: *N*-methyl-D-aspartic acid
NR: NMDA receptor subunit
RM: reference memory
ROI: region of interest
TNF: tumor necrosis factor
WM: working memory
